# Single-cell RNA-sequencing identifies various proportions of excitatory and inhibitory neurons in cultured human fetal brain cortical tissues

**DOI:** 10.3389/fnins.2023.1177747

**Published:** 2023-06-28

**Authors:** Rong Liu, Wei Dong, Dan Xiong, Lanqi Hu, Haoran Zhang, Xiaoping Yuan, Zhonghui Tang, Fang Fu, Xin Yang, Xia Wu

**Affiliations:** ^1^Zhongshan School of Medicine, Sun Yat-sen University, Guangzhou, Guangdong, China; ^2^Department of Prenatal Diagnostic Center, Guangzhou Women and Children’s Medical Center, Guangzhou Medical University, Guangzhou, Guangdong, China

**Keywords:** fetal brain, prefrontal cortex tissue, neuron progenitor cells, excitatory neurons, inhibitory neurons

## Abstract

**Introduction:**

Cortical neural progenitor cells possess the capacity to differentiate into both excitatory and inhibitory neurons. However, the precise proportions in which these progenitor cells differentiate remain unclear.

**Methods:**

Human fetal prefrontal cortical tissues were collected at various fetal stages and cultured in vitro. Bulk and single-cell RNA sequencing techniques were employed to analyze the resulting neuronal cell types, cell proportions, and the expression levels of cell-type marker genes.

**Results:**

The culture of fetal prefrontal cortex tissues obtained at gestation weeks 11 and 20 predominantly consisted of excitatory and inhibitory neurons, respectively. This abrupt transition in cell proportions was primarily driven by the differential lineage specificity of neural progenitors in the fetal cortical tissues at distinct stages of fetal brain development. Additionally, it was observed that the transcriptional profiles of cultured fetal cortical tissues were strongly influenced by the presence of FGF2.

**Discussion:**

This study presents a novel strategy to obtain excitatory and inhibitory neuronal cells from the culture of fetal cortical tissues. The findings shed light on the mechanisms underlying neurogenesis and provide an approach that might contribute to future research investigating the pathophysiology of various neural disorders.

## 1. Introduction

The human neocortex consists of about 170 million neurons (Kelley and Pasca, 2022), which are generated through the differentiation of neural progenitor cells (NPC) located in the cerebral cortex during vertebrate brain development. NPC develop into proliferative intermediate progenitor cells (IPC), which further differentiate into glutamatergic excitatory neurons (EN) and GABAergic inhibitory neurons (IN; Anderson et al., 1997, 2001; Nowakowski et al., 2017; Telley and Jabaudon, 2018). In addition, previous research has shown that NPC also differentiates into proliferative glial cells, including astrocytes and oligodendrocytes (Gorski et al., 2002). A recent single-cell RNA-sequencing (scRNA-seq) study by Zhong et al. on human fetal prefrontal cortex (PFC) tissues at gestational weeks (GW) 8 to 26 has shown that the NPC protein markers PAX6 and SOX2 were expressed at high density in the ventricular zone (VZ) as early as at GW8. This study further reported the sequential differentiation of NPC into neurons in the subventricular zone (sVZ) by GW12, and into oligodendrocytes and astrocytes in outer sVZ by GW23 (Zhong et al., 2018). Meanwhile, IN cells are differentiated from IN progenitor cells in caudal ganglionic eminence (CGE), medial ganglionic eminence (MGE), and preoptic area (POA), which migrate into the cortex in the later stage of fetal brain development (Delgado et al., 2022). Moreover, recent studies also showed that NPC in sVZ generates IN cells (Delgado et al., 2022). Collectively, the developing embryo brain generates different types of neuronal and/or non-neuronal cells during the increasing gestational time. Therefore, it is important to investigate whether *in vitro* culture of the embryonic PFC tissues at different gestational ages results in different proportions of cell types, which remains largely unknown.

The *in vitro* cultures of brain tissues have been previously applied to investigate neuronal cell types and functions. *In vitro* cultures of human embryonic spinal nerves led to defining the presence of acetylcholinergic and GABAergic neurons in 1985 (Kato et al., 1985), and fetal central neurons were cultured in 1995 (Sah, 1995). In recent years, the *in vivo* and *in vitro* cultures of adult neurogenesis have largely focused on the temporal and extra-temporal sVZ and the hippocampus, but *in vitro* cultures of the PFC, which contains all kinds of EN and IN and is the main information processing center, remains largely unexplored (Nogueira et al., 2022). Specifically, the effects of gestational ages on cell proportions in the human cultures of PFC tissues are largely unknown. In this study, we aimed to address this gap in knowledge by culturing human fetal brain cortical tissues at different gestational weeks and performing bulk RNA-seq and scRNA-seq to identify cell types, cell proportions, and genome-wide transcription levels. This study focused on two main topics: (i) the cell types that PFC tissue culture differentiates into at different gestation weeks, and (ii) the influences of fetal stages on cell proportions.

## 2. Materials and methods

### 2.1. Ethics statement

The human embryo collection and research analysis were approved by the Reproductive Study Ethics Committee of Guangzhou Women and Children’s Medical Center (No. 2021072816053867) and were performed in line with the principles of the Declaration of Helsinki. Informed consent for fetal tissue procurement and research were obtained from the patients after their decision to legally terminate their pregnancy but before the abortive procedure. All protocols followed the “Interim Measures for the Administration of Human Genetic Resources” administered by the Chinese Ministry of Health.

The C57BL/6 mice were purchased from the Laboratory Animal Center at Sun Yat-sen University. All procedures related to animals were performed following the ethical guidelines of Sun Yat-sen University. The use of animals in this study was approved by the Animal Care and Use Committee of Sun Yat-sen University (No. SYSU-IACUC-2020-070).

### 2.2. *In vitro* culture of human fetal prefrontal cortical brain tissues and mouse P0 cortical brain neurons

Human fetal PFC tissues and P0 mouse prefrontal cortical brain tissues were cultured following Brewer’s protocols (Brewer and Torricelli, 2007) with some simplification. Briefly, PFC tissues were dissected and collected in ice-cold HBSS embryo buffer (HBSS EB) consisting of HBSS, no calcium, and no magnesium (Invitrogen, 14,170,112) supplemented with 1 mM HEPES (Invitrogen, 15630080) and 1% Penicillin–Streptomycin (Invitrogen, 15140122). The PFC tissues were placed under a stereomicroscope in a laminar flow hood and the meninges and blood vessels were peeled off by tweezers. The HBSS EB buffer was discarded before around 500 μL fresh cold HBSS EB was added to the PFC tissues. Then the PFC tissues were cut into 0.3–0.5 mm mince and transferred into a 15-mL tube to centrifuge at 70 g for 20 s at room temperature (RT). After discarding the supernatant, a volume of 1-2 mL Papain (Worthington, LK003178) in HBSS buffer (Invitrogen, 14025092) with 10 U/μL DNase I (Worthington, LK003172) was then added to digest the PFC tissues in a 37°C oven with 10 round per minutes (rpm) shaking for 30 min at 30°C. The digested tissues were centrifuged at 500 g for 5 min at RT. The supernatant was discarded as much as possible before the cell pellets were resuspended in 2–3 mL HABG buffer, which contained neurobasal medium (Invitrogen, 21103049), 1× B27 (Invitrogen, 17504044), 1× GlutaMAX supplement (Invitrogen, 35,050–061). We then passed the digested tissues gently through a 1 mL low-bind tip 15 times in 45 s and settled down the cell suspension for 2 min to allow cell clusters and undigested tissues to sediment to the tube bottom. The single cells in the upper layer were then transferred to a new 15-mL tube. We repeated these processes 2–3 times to release as many single cells as possible from the digested tissues. The cell suspension was then centrifuged at 800 g for 5 min at RT to pellet the single cells. The supernatant was removed before the cell pellet was re-suspended in the HABG culture medium, which was comprised of HABG buffer supplemented with 1% Penicillin–Streptomycin (Invitrogen,15,140,122). The cells were seeded with 0.5–1 million/well in 6 well plates pre-coated with 0.1 mg/mL Poly-D-Lys (Sigma, 9866) dissolved in sterile miliQ water. For immunofluorescent staining, 20,000–30,000 cells were seeded in each well of a 48-well plate, each well of which was pre-loaded with a coverslip/well and was pre-coated with 0.1 mg/mL Poly-D-Lys dissolved in sterile miliQ water. Cells were cultured in a 37°C incubator with 5% CO_2_. A final concentration of 5 ng/mL FGF2 (Invitrogen, 13,256–029) was added into the HABG culture medium of the needed groups. Cell states were checked every day and the HABG culture medium was half replaced every 2 days since day 5. Cells were harvested cells on the indicated time points. The P0 mice PFC tissues were cultured for 10 days.

### 2.3. Immunocytochemistry

To conduct immunocytochemistry, the cells were rinsed once with RT PBS and then cross-linked with 4% PFA (Sangon Bioteh, E672002-0100) for 10 min at RT. The cross-linked cells were washed twice with ice-cold PBS, permeabilized, and blocked in PBS supplemented with 10% normal goat serum (NGS), 0.1% BSA, and 0.3% Triton X-100 for 1 h at RT. The cells were then incubated overnight at 4°C with primary antibodies diluted in PBS with 0.5% goat serum, and 0.3% Triton X-100. We used primary antibodies against MAP2 (Chicken, 1:1,500; Abcam ab5392; RRID: AB_2138153), GABA (Rabbit, 1:500; sigma, A2052: RRID: AB_477652), and NEUROD2 (rabbit, 1:300; Abcam, ab104430; RRID: AB_10975628). After primary antibody incubation, cells were washed 3 times with PBS and then incubated with secondary antibodies in PBS with 0.5% goat serum, 5 μg/mL DAPI, and 0.1% Triton X-100 for 1 h at RT. The secondary antibody Alexa Fluor Dyes (Invitrogen) 488 goat anti-rabbit (A11034; RRID: AB_2576217), 555 goat anti-mouse (A21424; RRID: AB_141780), and 647 goat anti-chicken (A21449; RRID: AB_1500594) were used at 1:1,000 dilution for amplifying the signal. The cells were then washed three times with PBS. Images were captured using a fluorescence microscope (Olympus, BX63). The number of NEUROD2^+^DAPI^+^MAP2^+^ and GABA^+^DAPI^+^MAP2^+^ cells were counted as EN and IN, respectively, from 3 to 4 fields of view and *P* was calculated by unpaired Student’s *t*-test.

### 2.4. Bulk RNA-seq

In this study, bulk RNA-seq analysis was performed with the primary cultured neurons from the GW11 PFC tissues at day 12, 14, 18, 20, and 23 and the neuronal cells from GW13, 15, and 20 PFC tissues at day 20 of culture. Briefly, at the indicated time points, we quickly washed the cells once with RT PBS and then added 1 mL of cold trizol (Invitrogen, 15,596,026) to the cells. After pipetting the mixture several times to release the RNA, we collected the sample to a 1.5-mL RNase-free tube and stored all the samples at −20°C until further processing. We extracted the total RNA from the collected samples according to the manufacturer’s manual and dissolved the RNA with 30–50 μL RNase-free water. We measured the RNA concentration by Qubit (Thermo Fisher Scientific, Q10210) and assessed the quality of the total RNA by measuring the RNA Integrity Number (RIN) score using the Qsep (Bioptic, Qsep1). All RNA samples displayed a RIN > 8, indicating high RNA quality. Finally, we constructed the RNA-seq library using the VAHTS TM Total RNA-seq (H/M/R) Library Prep Kit for Illumina kit (Vazyme, NR603) following the manufacturer’s protocol. GW11 and GW20 samples included 2 biological replicates and others included 2 technical replicates. All libraries were sequenced using Illumina Novaseq under the paired-end 150 bp model.

### 2.5. Analysis of the bulk RNA-seq data

**Figure 1:**
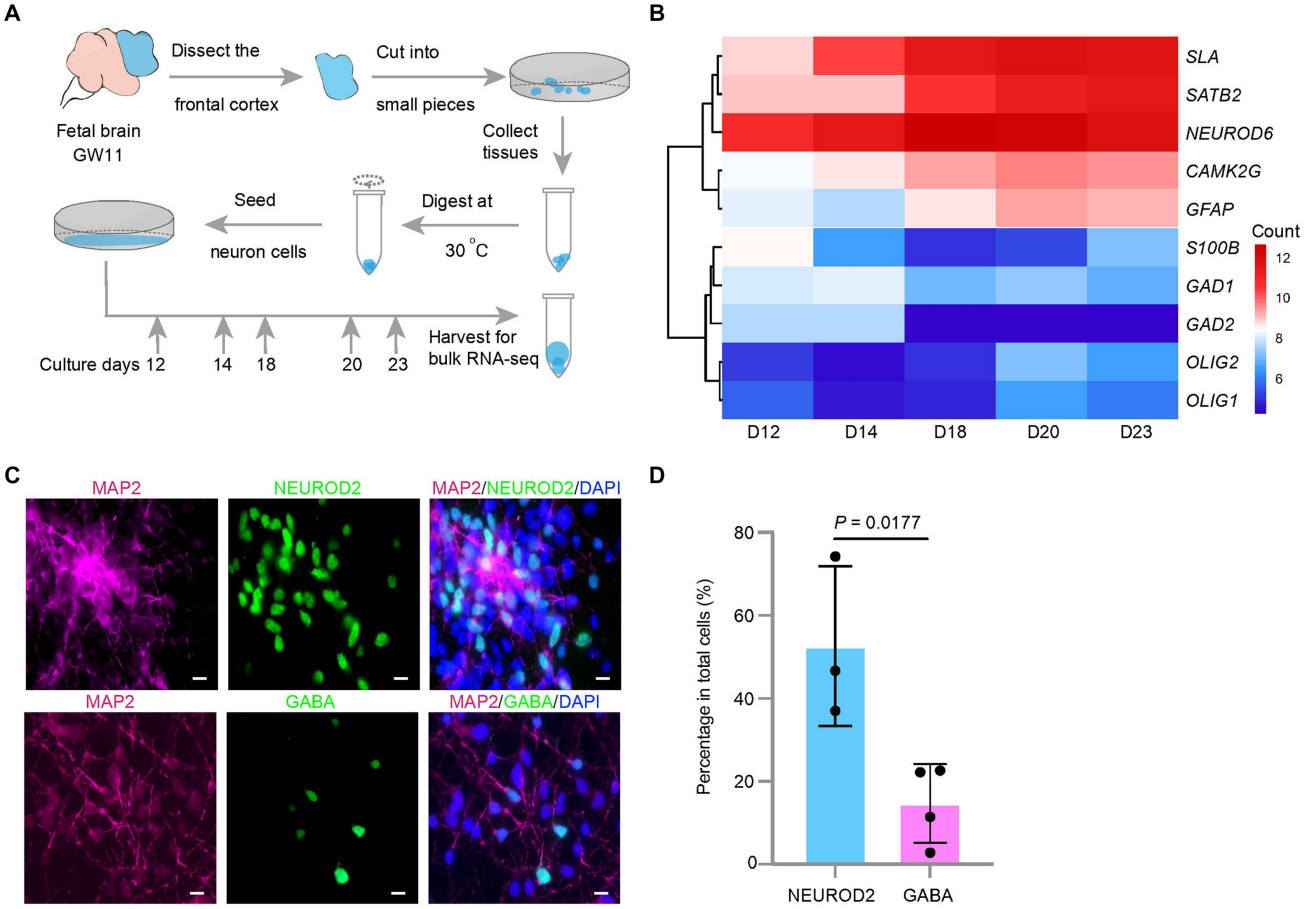
Effect of culture days on the transcription levels of excitatory neuron maker genes in the culture of the fetal cortex tissues at GW11. **(A)** Experimental schematic for bulk RNA-seq in primary cultured neurons at different culture days. **(B)** Heatmap displays differentially expressed maker genes in cultured GW11 PFC tissues at day 12, 14, 18, 20, and 23. Color bar denotes the normalized read counts representing gene expression levels. **(C)** Immunofluorescence staining for protein markers of pan neurons (MAP2), excitatory neurons (NEUROD2), and inhibitory neurons (GABA) in GW11 PFC tissue culture at day 20. Scale bars, 10 μm. *n* = 3 independent replicates. **(D)** Quantification of the proportions of NEUROD2^+^ excitatory neurons and GABA^+^ inhibitory neurons in primary cultured neurons after 20 days *in vitro* culture of GW11 PFC tissues. Each point represents one field of view. Data are present as mean ± SD. *P* was calculated by unpaired Student’s *t*-test.

**Figure 2:**
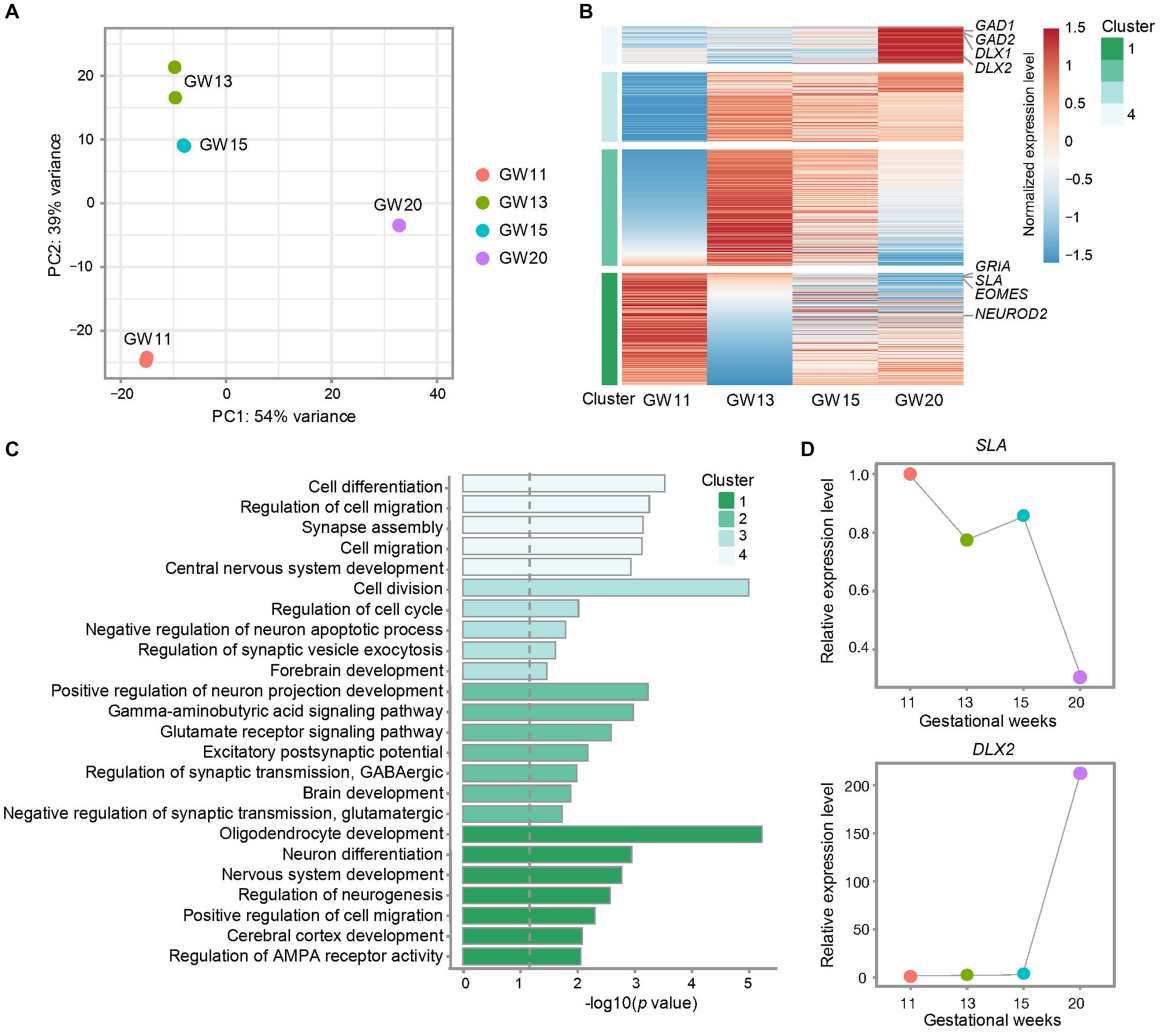
Effects of different gestational ages on the transcription level of inhibitory neuron maker genes in the culture of the fetal cortex tissues. **(A)** Principal component analysis (PCA) for bulk RNA-seq data from cultures of PFC tissues at GW11, 13, 15, and 20. Each point represents a replicate. **(B)** Heatmap shows the differentially expressed genes in the cultures of PFC tissues at different gestational ages. The relative expression level of each gene is normalized to −1.5 to 1.5 by z-score. Representative genes are listed on the right side. All genes are divided into four clusters. **(C)** Gene ontology (GO) enrichment for the differentially expressed genes from the four clusters of genes defined in **(B)**. The dashed gray line indicated the significance *p* = 0.05. **(D)** Relative expression levels of marker genes for EN (*SLA*) and IN (*DLX2*) in the cultures of PFC tissues at GW11, 13, 15, and 20.

The clean RNA-seq reads were mapped to the human reference genome (hg38) or mouse reference genome (mm10) using Hisat2 (Kim et al., 2019) with default parameters. Then, high-quality mapped reads were extracted using Samtools (Li et al., 2009) with the “-q 20” parameter setting and gene expression raw read counts were estimated by HTseq (Anders et al., 2015). The statistics during raw data processing are shown in Tables 1–3 for the bulk RNA-seq data used for Figures 1, 2. To clarify the causal factor for the phenotypic differences among primary neurons cultured from GW11, 13, 15, and 20 PFC tissues cultured primary neurons, we performed differentially expressed analysis with the DESeq2 R package (Love et al., 2014). PCA analysis was performed using “plotPCA” function of DEseq2 (Figure 2A). The parameters were “| log2(fold change) | > 1, FDR < 0.05” for identifying differentially expressed genes among 4 groups. We obtained a total of 4,849 significant differentially expressed genes. We then used the SOM package to classify the gene expression patterns in the cultured primary neurons of GW11, 13, 15, and 20 PFC tissues into four categories. We identified 1,619, 1,678, 1,015, and 537 significant differentially expressed genes for Cluster 1, Cluster 2, Cluster 3, and Cluster 4, respectively. The normalized count matrices were used to plot selected gene expressions using the pheatmap R package (Figure 2B). Gene ontology (GO) functional enrichment of differentially expressed genes was conducted using the DAVID web tool^1^ (Huang da et al., 2009; Sherman et al., 2022; Figure 2C).

**Table 1:** Statistics of the RNA-seq data for cultured GW11 PFC tissues at days 12, 14, 18, 20, and 23.

Sample name	Assay	Genome	Clean reads number	Sorted reads number	Unique reads number	Valid reads number	Total gene number	Detected gene number (count > 0)	Detected gene (count > 0) percentage	Genes (count > 10) number	Genes (count > 10) percentage
GW11_D12	RNA-seq	hg38	46,143,082	43,683,352	42,864,666	92.90%	60,617	26,357	43.48%	16,442	27.12%
GW11_D14	RNA-seq	hg38	56,227,430	53,502,566	52,651,384	93.64%	60,617	27,726	45.74%	17,237	28.44%
GW11_D18	RNA-seq	hg38	43,434,754	41,352,832	40,706,450	93.72%	60,617	26,722	44.08%	16,358	26.99%
GW11_D20	RNA-seq	hg38	55,209,342	52,072,932	50,970,470	92.32%	60,617	28,467	46.96%	18,320	30.22%
GW11_D23	RNA-seq	hg38	38,500,092	35,997,406	35,304,168	91.70%	60,617	27,094	44.70%	16,586	27.36%

**Table 2:** Statistics of the RNA-seq data for the cultured fetal PFC tissues at GW11, 13,15, and 20.

Sample name	Assay	Genome	Clean reads number	Sorted reads number	Unique reads number	Valid reads number	Total gene number	Detected gene number (count > 0)	Detected gene (count > 0) percentage	Genes (count > 10) number	Genes (count > 10) percentage
GW11_D20	RNA-seq	hg38	38,517,744	36,441,630	35,876,472	93.14%	60,617	26,169	43.17%	15,776	26.03%
GW11_D20	RNA-seq	hg38	55,209,342	52,072,932	50,970,470	92.32%	60,617	28,467	46.96%	18,320	30.22%
GW13_D20	RNA-seq	hg38	48,876,920	46,056,562	45,402,896	92.89%	60,617	26,372	43.51%	16,201	26.73%
GW15_D20	RNA-seq	hg38	45,371,070	42,366,248	41,679,544	91.86%	60,617	26,102	43.06%	16,427	27.10%
GW20_D20	RNA-seq	hg38	55,105,674	50,862,216	49,902,184	90.56%	60,617	28,334	46.74%	18,513	30.54%
GW20_D20	RNA-seq	hg38	38,394,284	35,745,268	35,134,246	91.51%	60,617	27,258	44.97%	16,905	27.89%

**Table 3:** Statistics of the RNA-seq data for the P0 mouse PFC tissue culture.

Sample name	Assay	Genome	Clean reads number	Sorted reads number	Unique reads number	Valid reads number	Total gene number	Genes (count > 0)	Genes (count > 0) percentage	Genes (count > 10)	Genes (count > 10) percentage
Mouse_P0_D10_FGF	RNA-seq	mm10	35,963,312	33,145,306	32,357,284	89.97%	55,421	24,349	43.93%	16,439	29.66%
Mouse_P0_D10_FGF	RNA-seq	mm10	39,292,636	36,590,038	35,722,256	90.91%	55,421	24,552	44.30%	16,689	30.11%
Mouse_P0_D10_noFGF	RNA-seq	mm10	39,447,254	36,447,338	35,538,628	90.09%	55,421	24,775	44.70%	16,683	30.10%
Mouse_P0_D10_noFGF	RNA-seq	mm10	40,748,052	37,723,300	36,763,096	90.22%	55,421	24,932	44.99%	16,717	30.16%
GW13_NOFGF	RNA-seq	hg38	51,167,034	47,686,126	46,852,984	91.57%	60,617	27,649	45.61%	17,131	28.26%
GW13_FGF	RNA-seq	hg38	48,876,920	46,056,562	45,402,896	92.89%	60,617	26,372	43.51%	16,201	26.73%

### 2.6. Preparation of single-cell suspension for single-cell RNA-seq

Single-cell suspensions for the scRNA-seq were prepared from the GW11 and GW20 PFC tissues culture on day 20. The cells were washed twice with PBS at RT and then digested into single cells using 1 mL 37°C pre-warmed trypsin. The cells were then collected into a 1.5-mL low-bind tube and centrifuged at 300 g for 5 min at RT. The cell pellet was resuspended in 1 mL PBS and passed a 35-μm filter. To remove cell debris, we adjusted the volume of the filtered cell suspension to 3.1 mL with PBS, added 0.9 mL debris removal solution (Miltenyi, 130–109-398) into the cells, and mixed well. A volume of 2 mL PBS was layered on top of the cells without disturbing the cells before centrifuging the cells at 3,000 g for 10 min at 4°C. The obtained cell pellet was rinsed once with 5 mL PBS. After that, we resuspended the cells in PBS supplemented with 0.04% bovine serum albumin (BSA) and conducted quality control to evaluate cell density (900–1,200 cells/μL) and the ratio of cell viability (>90%), cell debris (<5%), and clusters (<3%).

### 2.7. Single-cell RNA-seq

Single-cell RNA-seq analysis was performed using the Chromium Next Single Cell 5′ Kit, Library & Gel Bead Kit v2 (10x Genomics, PN-1000265) following the manufacturer’s protocols. Briefly, a total of 16,000 prepared single-cell suspensions were loaded into Chip K to achieve a recovery of approximately 10,000 cells per run. After reverse transcription, cDNA cleanup, and amplification, quality control was performed using the Qubit HS kit and Qsep to measure the cDNA concentration and size distribution, respectively. For library construction, 50 ng of cDNA was fragmented, end-repaired, ligated to adapters, and underwent indexed PCR amplification. The library was then double-size selected using 0.6 × −0.8 × volume of SPRI-selected beads (Beckman, B23318). The purified libraries were sequenced using Illumina Novaseq under the model paired-end 150 bp.

### 2.8. Analysis of scRNA-seq data

The scRNA-seq data of each sample were aligned to the human reference genome (GRCh38) using CellRanger pipelines (version 5.0.0). Aligned feature-barcode count matrices were generated using the “cellranger count” command with default parameters. The pre-filtered gene expression matrices were then imported into the R Seurat package (version 4.0.6; Hao et al., 2021) for downstream analysis. Briefly, cells containing <200 genes and genes expressed in <3 cells of the data were removed, and low-quality cells with >20% of UMIs derived from the mitochondria were also excluded from further analyses. Ambient RNA was removed from the cells by SoupX (Young and Behjati, 2020) with the contamination fraction parameter setting “conFrac = 0.2.” To avoid the dominant effect of mitochondria and ribosomal protein-coding genes, which usually show very abundant expression levels in subpopulations of cells, these genes were also removed from the subsequent analysis. Furthermore, doublets were predicted using the DoubletFinder (McGinnis et al., 2019) R package, and were then excluded for downstream analysis. After quality control and cell filtering, pre-processed gene expression matrices were then normalized by the “NormalizedData” function. A total of 2,000 most variable genes were identified for each sample using the “FinderVariableFeature” function with the “vst” method. The variable genes from each sample were combined to obtain a unique list of the highly variable genes. Subsequently, the normalized gene expression matrixes from each sample were merged, and the “ScaleData” function was used to scale and center the whole gene expression matrix. To reduce the dimensionality, PCA was conducted on the unique variable gene list to identify the top 30 principal components using the “RunPCA” function in Seurat. To eliminate the batch effects from different samples, we used the Harmony (Korsunsky et al., 2019) to integrate two datasets into a shared space for unsupervised clustering. Briefly, we fed the calculated PCA matrix into the “RunHarmony” function implemented in Seurat. As the Harmony algorithm can integrate categorical covariates, we set the samples and culture conditions as two covariates for batch effect correction with the corresponding theta parameters set as 2 and 1, respectively. Next, the batch-effect-corrected Harmonoy embeddings were applied to dimension reduction and visualization using the “RunUMAP” function with the first 20 principal components. The expression levels of representative genes were plotted with Uniform Manifold Approximation and Projection (UMAP). Finally, the “FindNeighbors” and “FindClusters” functions were used to perform cell clustering, with the parameter setting of “reduction = “harmony,” dims = 1:20, resolution = 0.6.” Differentially expressed genes in each cluster and between different samples were identified by the “FindAllMarkers/FindMarkers” functions in Seurat using the “MAST” test algorithm (Finak et al., 2015), with the parameter setting of “test.use = “MAST,” min.pct = 0.25, logfc.threshold = 0.5.” The statistics for the raw scRNA-seq data processing are included in Supplementary Table S1, and the quality control plots were displayed in Supplementary Figure S1. Cell type annotation was performed based on the expression patterns of canonical neuron-specific cell type markers and reference mapping with known cell-types in the reference datasets (Delgado et al., 2022), and contaminated cells lacking distinct markers of known cell types were further excluded.

### 2.9. Inferring cell differentiation tendency

To display the cell type differentiation of EN and IN in GW11 and GW20 more intuitively, we inferred the cell differentiation direction by the URD package (Farrell et al., 2018). Briefly, the first 20 principal components of batch-effect-corrected Harmony embeddings were used to obtain 2-dimensional t-SNE embeddings by applying the “RunTSNE” functions in Seurat. We imported the information in the obtained Seurat object into the URD package after removing the oligodendrocytes and astrocytes. Transition probabilities were then calculated using “calcDM” function based on the harmony embeddings. The obtained transition probabilities overlaid with TSNE embeddings (van der Maaten and Hinton, 2008) were visualized using the “plotDim” function.

## 3. Results

### 3.1. Culture days influence transcription levels of marker genes for excitatory neurons in primary cultures of prefrontal cortex tissues at early weeks of gestation

NPC emerges in the prefrontal lobe of the embryonic brain in early developmental stages. This poses two questions: (1) will the NPC continue to proliferate or differentiate into neurons after culturing? (2) If NPC differentiates, which neuron type will be preferential? To investigate these questions, early brain PFC tissues were obtained at GW11. After 12, 14, 18, 20, and 23 days of culture, the cell number increased to 8–10 times the seeded cell number. These cells were collected for transcriptome analysis using bulk RNA-seq (Figure 1A). The results showed that the transcription levels of the marker genes for EN, *NEUROND6*, *SATB2*, *CAMK2G*, and *SLA* increased with increasing culture days, and peaked at day 20 (Figure 1B). Maker genes for IN (*GAD1* and *GAD2*) and oligodendrocytes (*OLIG1* and *OLIG2*) showed low transcription levels through the culture period (Figure 1B). In contrast, astrocytes marker genes *GFAP* showed transcriptional activity in the culture system, indicating their emergence. Immunostaining of the cells at day 20 confirmed the higher ratio of EN (NEUROD2) compared to IN (GABA) (Figures 1C,D). Taken together, EN arose in the *in vitro* culture of the GW11 fetal cortex tissues and the transcription levels of EN marker genes peaked at day 20.

### 3.2. The gestation time affects transcription levels of inhibitory neuron marker genes in primary cultures of prefrontal cortex tissues

The human brain undergoes critical biological processes and a prolonged period of development and maturation (Delgado et al., 2022). Therefore, to figure out whether NPC at different gestational weeks differentiates into varied neuronal cell types, we explored the cell ratios in the culturing of PFC tissues from different gestational ages. To this end, we cultured primary human PFC tissues obtained at GW11, 13, 15, and 20 for 20 days. Bulk RNA-seq was performed with two replicates for each time point. Principal component analysis (PCA) confirmed high data repeatability for each sample. The primary cultured NPC neurons displayed notable differences between samples from different gestation weeks, with the highest similarities between GW13 and GW15 and the largest difference between GW11 and GW20 (Figure 2A), indicating an intrinsic alteration between the cultures of GW11 and GW20 samples. Differential expression analyses among the samples from the four gestation time points determined four clusters of differentially expressed genes. The result revealed that marker genes for IN (*GAD1*, *GAD2*, *DLX1*, and *DLX2*) exhibited the highest transcription levels in the GW20 sample, while marker genes for EN (*SLA*, *GRIA*, and *NEUROD2*) peaked in GW11 cells (Figure 2B). Pathway enrichment analysis showed that genes highly transcribed at GW11 in Cluster 1 were enriched in pathways such as cerebral cortex development, positive regulation of cell migration, and regulation of neurogenesis. Genes in Cluster 2 were mainly related to pathways about positive regulation of neuron projection development, brain development, and excitatory postsynaptic potential. Genes in Cluster 3 were mainly enriched in pathways related to the regulation of cell division, negative regulation of neuron apoptotic process, and forebrain development. GW20 differentially highest expressed genes in Cluster 4 displayed enrichment of pathways like cell differentiation, regulation of cell migration, synapse assembly, cell migration, and central nervous system development (Figure 2C). After the culture for 20 days, the transcription level of the inhibitory IPC marker gene *DLX2* increased almost 200-fold in GW20 cells compared to that in GW11, 13, and 15 tissues. Meanwhile, the expression of the EN marker genes *SLA* showed a continuous decreasing trend along the gestation time of the cortex tissues (Figure 2D). These results indicated that different types of neuronal cells emerged in the culture of cortex tissues from different gestation time points, with transcription levels increasing for the IN-marker genes and decreasing for the EN-marker genes along the gestation age.

### 3.3. Single-cell RNA-seq revealed different proportions of excitatory and inhibitory neurons in cultures of fetal cortex tissues with different gestational ages

**Figure 3:**
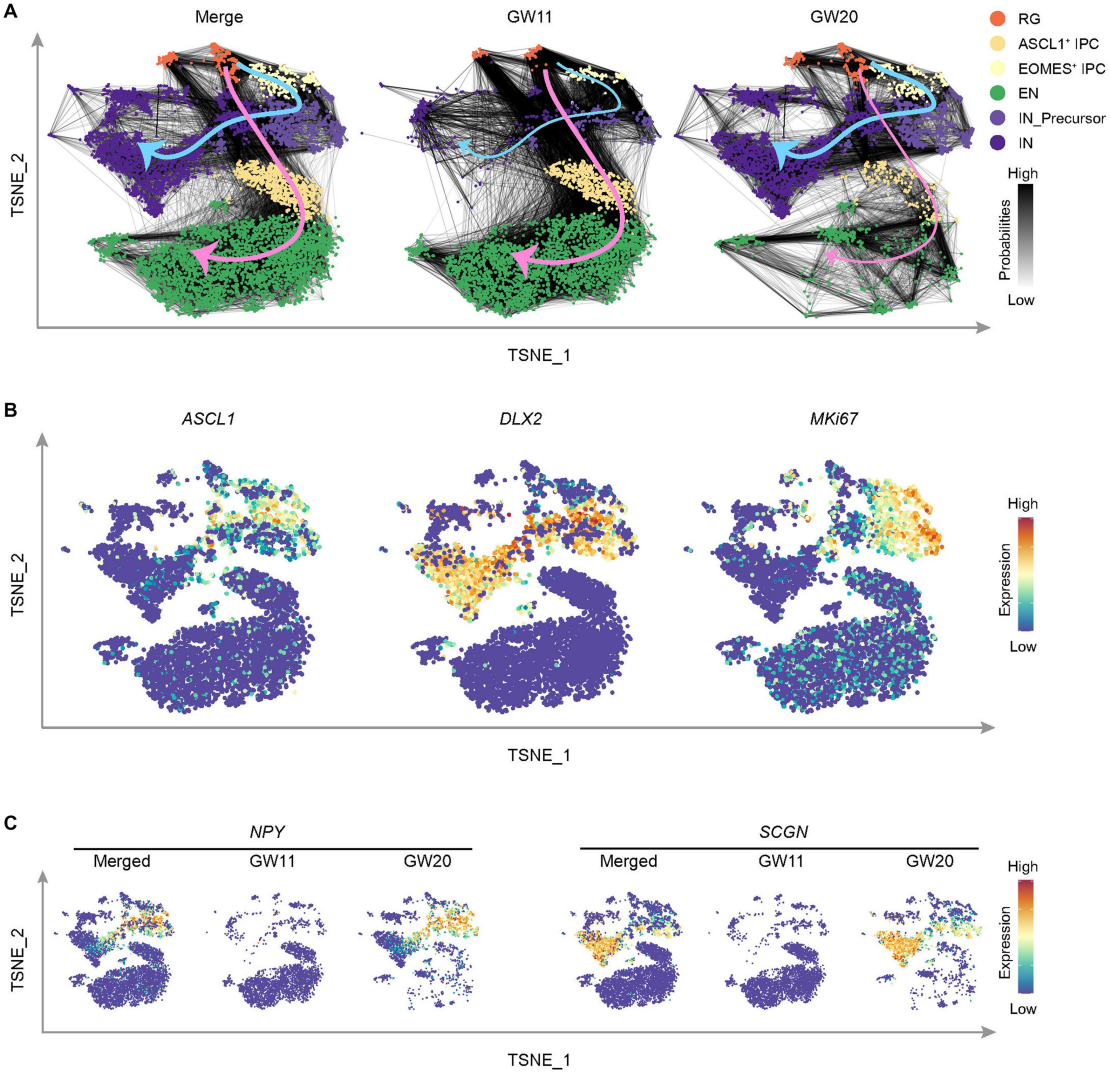
Single cell RNA-seq analysis of the cultured fetal cortex tissues at GW11 and GW20. **(A)** Uniform Manifold Approximation and Projection (UMAP) visualization shows eight cell clusters in the scRNA-seq data of cultured fetal PFC tissues at GW11 and GW20. Each dot represents a cell. Each cell cluster is colored by cell type. RG, radial glia; ASCL1^+^ IPC, proliferating intermediate neural progenitor cells expressing *ASCL1*; EOMES^+^ IPC, *EOMES* positive intermediate progenitor cells; EN, excitatory neurons; Astro, astrocytes; Oligo, oligodendrocytes; IN_Precursor, inhibitory neuron precursor cells; IN, inhibitory neurons. **(B)** Single-cell transcription levels of representative genes illustrated in the UMAP plot from **(A)**. Transcription levels are color-coded: gray, not expressed; blue, expressed. **(C)** Dot plot represents the relative average expression of marker genes for each cluster from the UMAP in **(A)**. As in the legend, the dot size denotes the proportion of cells that expressed the gene in each cluster, and the dot color indicates the average expression level of the gene in each cluster. **(D)** Differentially expressed genes in each cluster in **(A)**. Each row denotes a gene, and each column represents a cell. The color bar represents the relative expression level. Representative genes are listed on the left. **(E)** Proportions of the cells in each cluster of **(A)**. **(F)** Differentially expressed genes between GW11 and GW20 samples within each cell cluster. Each dot represents a gene. Genes differentially higher expressed in GW11 and GW20 samples in each cluster are colored in red and green, respectively; while the genes not significantly (NS) differentially expressed between the two samples are gray colored. Representative differentially expressed genes are listed at the top and bottom of each cluster, respectively. Dash lines represent the abs [log2(fold change)] = 0.5.

With the significant elevation of EN marker gene expression levels in the culture of GW11, 13, and 15 PFC tissues and of IN marker genes in GW20 PFC tissues, we investigated the underlying rationale for this alteration. To this end, we cultured GW11 and GW20 PFC tissues until day 20 and dissociated the samples into single-cell suspension, which was subjected to scRNA-seq analysis. A total of 4,613 GW11 and 4,638 GW20 cells passed quality control and were integrated after removing batch effects by Harmony (Korsunsky et al., 2019). Cell clustering and UMAP visualization of the integrated datasets revealed eight cell clusters in each of the cultures of both GW11 and GW20 PFC tissues. These clusters included radial glia (RG), IPC expressing *EOMES* (EOMES^+^ IPC), IPC expressing *ASCL1*(ASCL1^+^ IPC), IN precursor cells (IN_Precursor), IN, EN, oligodendrocytes, and astrocytes (Figure 3A). Cells in each cluster specifically expressed high levels of cell-type marker genes, such as *HOPX* and *VIM* for RG, *EOMES* for EOMES^+^ IPC, *MKI67* and *ASCL1* for ASCL1^+^ IPC, *MKI67* and *GAD2* for IN_precursor, *OLIG1* and *OLIG2* for oligodendrocytes, *SLC1A3* for astrocytes, *NEUROD2* and *NEUROD6* for EN, and *GAD1* and *GAD2* for IN (Figures 3B–D). These cell-type marker genes were among the top 20 differentially expressed genes in each cluster (Figure 3D), confirming the reliability of the cell clustering and annotation.

We then explored the impact of gestational ages on the proportions of cell types present in the cultures of GW11 and GW20 PFC tissues. The results revealed substantial differences in the cell ratios between these two samples, even though both contained all eight identified clusters. The most prevalent cell type in the GW11 sample was EN, which accounted for 68.1% of the cells present (3,140 cells). In contrast, the GW20 samples possessed only 630 EN cells, representing only 13.6% of the total cell population. In the GW20 PFC tissue culture, IN was the dominant cell type, comprising 49.3% of the whole sample (2,287 cells), while only 250 IN (5.4% of the sample) were found in the GW11 culture (Figure 3E). These findings were consistent with higher expression levels of EN and IN marker genes in GW11 and GW20 PFC tissue cultures, respectively. Interestingly, the EOMES^+^ IPC displayed a higher ratio in the GW11 (14.0%) sample compared to GW20 (4.9%; Figure 3E). Such IPC has been shown to differentiate into EN cells (Ziffra et al., 2021). In contrast, IN_Precursor presented a ratio of 15.6% of the total cells (723 cells) at GW20 but of only 4.8% (220 cells) at GW11. These findings could explain the higher ratio of EN and IN in the GW11 and GW20 cultures, respectively. Taken together, our results showed that cultures of PFC tissues at GW11 and GW20 generated primary EN and IN, respectively, resulting in a signification increase in the expression levels of their respective maker genes.

Since the eight cell types arose in both the cultures of PFC tissues at GW11 and GW20, we then investigated the difference within each cell type that originated from PFC tissues at different gestation time points. The comparison between astrocytes and oligodendrocytes, which have <100 cells in GW11 or GW20, was considered non-significant. Despite the high resemblance of cells of each cell type from the two gestation ages, differential expression analysis revealed multiple differentially expressed genes within each cell type. For instance, EN cells in GW11 PFC tissue culture expressed higher levels of *BCL11B* and *GRIA1*, which were related to excitatory neuron differentiation (De Bruyckere et al., 2018) and excitatory synaptic function (Ji et al., 2020), respectively. In contrast, IN cells in the culture of GW20 PFC tissue showed a higher transcription of *DLX1* and *DLX2*, which is involved in IN cell differentiation (Delgado et al., 2022). Notably, progenitor cells (RG, EOMES^+^ IPC, and ASCL1^+^ IPC) in the GW11 sample displayed a higher expression level for genes regulating progenitor cell proliferation and differentiation, such as *SOX5* and *GLI3* (Martinez-Morales et al., 2010; Wang et al., 2011). On the contrary, progenitor cells (RG, ASCL1^+^ IPC, and IN_Precursor) in the GW20 sample expressed higher levels of genes enhancing IN differentiation, like *DLX1*, *DLX2*, and *ASCL1* (Kessaris et al., 2014; Figure 3F). Our results distinguished expression differences within the same cell types in the culture of PFC tissues at different gestation time points.

Taken together, the scRNA-seq analysis revealed that the culture of GW11 and GW20 PFC tissues give rise to predominant EN and IN cells, respectively, which is consistent with the higher expression levels of their respective marker genes in the culture of the PFC of the two different gestation timepoints.

### 3.4. Cell differentiation analysis reveals a differential differentiation of neural progenitor cells in the cultures of cortex tissues at different gestational time points

**Figure 4:**
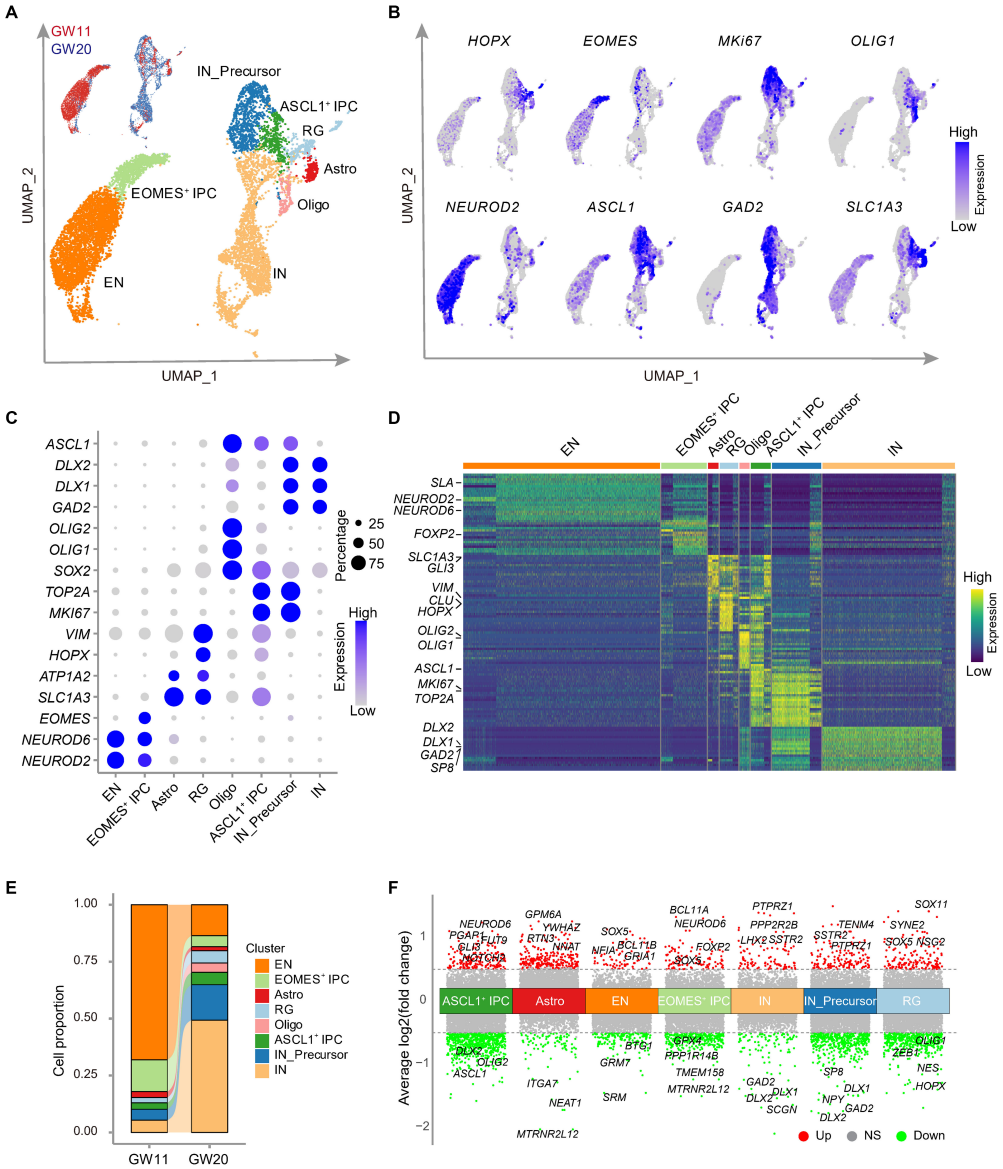
Cell differentiation tendency in the cultured fetal cortex tissues at GW11 and GW20. **(A)** T-distributed stochastic neighbor embedding (TNSE) plots show the single cell clusters with the transition probabilities between cells in merged (left), GW11 (middle), and GW20 (right) samples. The cell clusters are defined as in this figure and colored as in the legend. The gray lines between cells indicate transition probabilities between the cells and the line color represents the probability level. The pink and blue arrows denote the cell transition tendencies for EN and IN cells, respectively. **(B)** Single-cell expression levels of representative genes illustrated in the merged TSNE plot from **(A)**. Transcription levels are color-coded: blue, not expressed; red, expressed. **(C)** Single-cell expression levels of representative CGE/MGE marker genes illustrated in the merged TSNE plot from **(A)**. Transcription levels are color-coded: blue, not expressed; red, expressed.

Based on the preferential differentiation of GW11 and GW20 PFC tissue cultures into respective EN and IN cells, it is unclear when and how this shift in cell proportion ratio develops. To address this question, we inferred the transition probabilities between cells using the scRNA-seq data sets of GW11 and GW20. The results displayed two clear directions for cell transition, with the EN direction from RG to EOMES^+^ IPC to EN and the IN direction through the way RG to ASCL1^+^ IPC to IN_Precursor to IN (Figure 4A). Both ASCL1^+^ IPC and IN_Precursor cells expressed *ASCL1*, a gene regulating the differentiation of oligodendrocytes and IN (Sudarov et al., 2011; Figure 4B). Moreover, IN_Precursor cells expressed both *DLX2* and *MKI67*, which might proliferate and differentiate into IN. Notably, the two directions were contributed differentially by the two samples. As in Figure 4A, GW11 samples exhibited much higher cell transition probabilities in the EN direction, with much more links connecting the larger numbers of EOMES^+^ IPC cells upward with RG and downward with EN in this sample than in GW20. In contrast, cells from GW20 PFC culture showed more evident cell transition probabilities in the IN direction, with most links occurring between RG-ASCL1^+^ IPC-IN_Precursor-IN cell clusters. This tendency is somehow attributed to the differential progenitor cell states in the cultures of PFC tissues of the two gestational time points, which displayed different transcription profiles (Figure 3F).

IN classically originated from CGE/MGE (Delgado et al., 2022). We then assessed whether cells migrated from CGE/MGE contributed to the differential cell differentiation trajectories and cell proportions in the cultures of PFC tissues in GW11 and GW20. Both CGE/MGE marker genes *NPY* and *SCGN* were almost absent in the GW11 sample (Figure 4C), indicating that the IN and IN_Precursor cells in this culture are differentiated from the PFC local neural progenitor cells. However, most IN_Precursor cells and IN in GW20 expressed *NPY* and *SCGN*, respectively (Figure 4C), alluding that most of these cells in the two clusters can be traced back to inhibitory neuronal progenitors migrated from CGE and MGE. Taken together, neural progenitor cells in the cultures of PFC tissues at GW11 and GW20 primarily differentiate into EN and IN possibly due to the differential progenitor cell states and cell origins in the PFC tissues at the two gestational time points.

### 3.5. Effect of FGF2 on primary cultures of pre-frontal culture tissues

**Figure 5:**
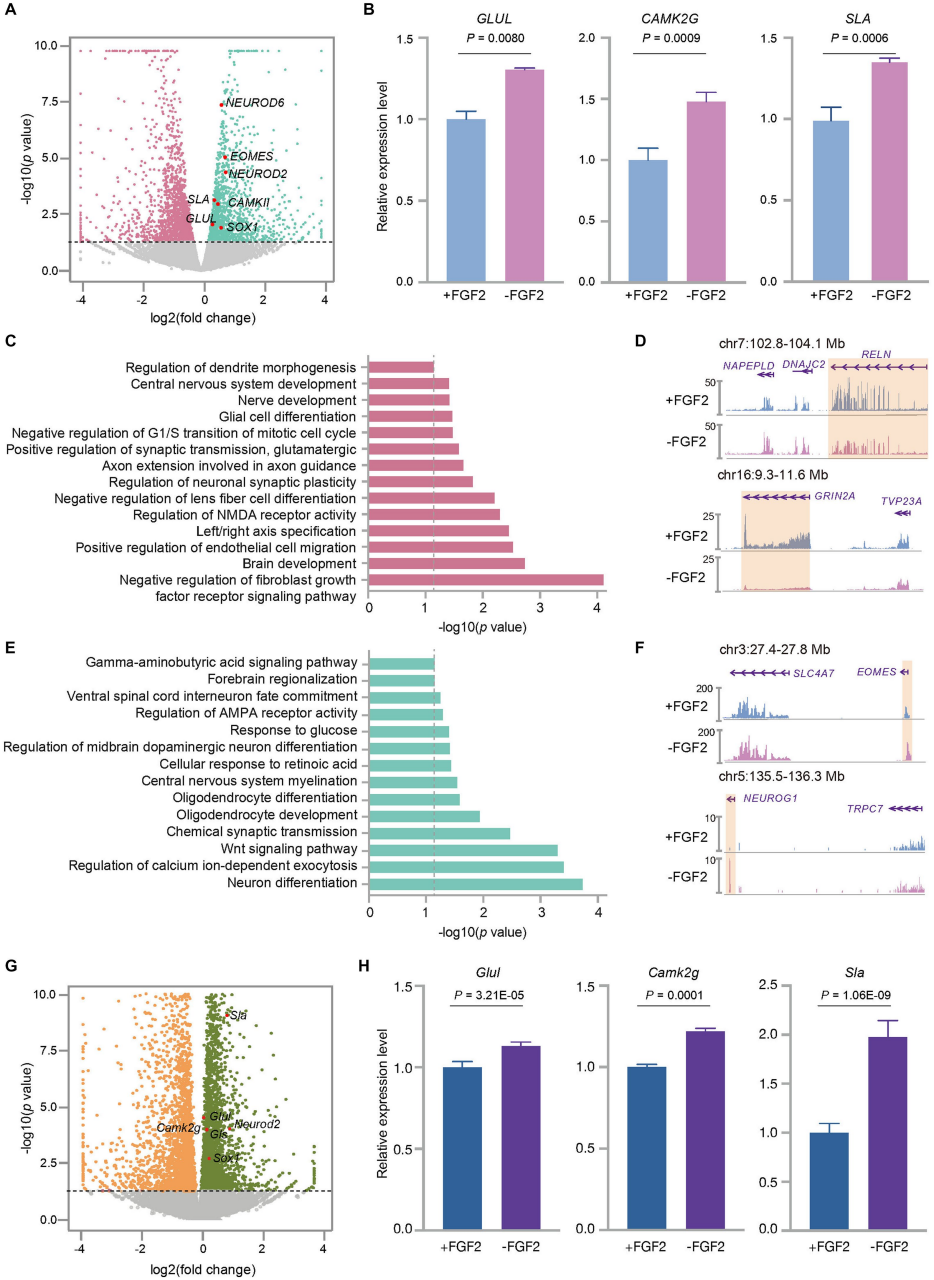
FGF2 influenced the expression profiles of the cultured fetal cortex tissue at GW13. **(A)** Volcano plot shows the differentially expressed genes between GW13 PFC tissues cultured with or without FGF2. Each dot represents a gene. Pink and green dots represent genes showing a higher transcription level in the culture with and without FGF2 administration, respectively. Gray dots indicate the genes that were not significantly changed by FGF2 treatment. The representative EN marker genes higher expressed in the culture without FGF2 are highlighted in red. **(B)** Relative expression levels of EN maker genes (*GLUL*, *CAMK2G*, *SLA*) in GW13 PFC tissue cultures with or without FGF2 administration. Data are present as mean ± SD; *n* = 2; and *P* was calculated with package DESeq2. **(C)** GO enrichment of genes with increased expression levels in the GW13 PFC culture with FGF2. The gray dashed line indicated the cutoff *p* = 0.05. **(D)** RNA-seq track views show the transcription levels of *RELN* and *GRIN2A* in GW13 PFC culture with or without FGF2. The orange boxes highlight the transcript regions of the genes. **(E)** GO enrichment of genes with decreased expression levels in the treatment with FGF2. The gray dashed line indicated the cutoff *p* = 0.05. **(F)** RNA-seq track views show the transcription levels of *EOMES* and *NEUROG1* in GW13 PFC culture with or without FGF2. The orange boxes highlight the transcript regions of the genes. **(G)** Volcano plot shows the differentially expressed genes between the cultures of P0 mouse brain tissues with or without FGF2. Each dot represents a gene. Orange and dark green dots represent genes with increased transcription levels in the culture with and without FGF2, respectively. Gray dots indicate the genes that were not significantly changed by FGF2 treatment. The representative EN marker genes higher expressed in the cultures without FGF2 are highlighted in red. **(H)** Relative expression levels of mouse EN maker genes (*Glul*, *Camk2g*, and *Sla*) in the cultures of P0 mouse brain with or without FGF2 administration. Data are present as mean ± SD. *n* = 2, and *P* was calculated with package DESeq2.

Fibroblast growth factor 2 (FGF2) is known to play a crucial role in promoting neural cell survival, migration, and differentiation (Hayashida et al., 2020). Previous studies have reported conflicting findings on the effects of FGF2 on neuronal cell culture (Qian et al., 1997; Woodbury and Ikezu, 2014), and its impacts on human fetal brain tissue cultures remain largely unknown. However, FGF2 has been shown to promote the proliferation of neural progenitor cells (Brewer and Torricelli, 2007; Gupta et al., 2018; Recabal et al., 2021) and increase the synaptic efficacy of excitatory neurons (Gupta et al., 2018) when added to the culture medium of human and mouse cortex tissues. To illustrate the effects of FGF2 on neuronal cell differentiation in the culture of human fetal PFC tissues, we cultured GW13 samples with and without FGF2. Our results showed that the treatment with FGF2 administration decreased the expression levels of *EN* marker genes *NEUROD6*, *GLUL*, *CAMK2G*, and *SLA* (Figures 5A,B). Further analysis of differentially expressed genes showed that culture with FGF2 upregulated a total of 592 genes, which were enriched in pathways related to brain development, synaptic transmission, and regulation of neuronal synaptic plasticity. Notably, neuromodulation related processes such as NMDA receptor activity, neuronal synaptic plasticity, and central neuron system development were also enriched in the FGF2 upregulated genes (Figure 5C). Additionally, genes like *RELN*, a regulator of neuronal stratification, microtubes function, and neuronal migration (de Guglielmo et al., 2022), and *GRIN2A*, a subunit of glutamate receptor (Santos-Gómez et al., 2020), were included in the FGF2- enhanced genes (Figure 5D). These findings were consistent with the known functions of FGF2 in neuronal cell migration and EN synaptic efficacy (Gupta et al., 2018; Hayashida et al., 2020). In contrast, FGF2 administration resulted in significant downregulation of 480 genes, which were enriched with terms like neuron differentiation and regulation of AMPA receptor activity (Figure 5E). Importantly, FGF2 attenuated the expression levels of genes related to EN and EN progenitor cell differentiation, like *NEUROG1* (Kim et al., 2011) and *EOMES* (Figure 5F), consistent with the term neuron differentiation in Figure 5E. Taken together, these findings suggested that FGF2 decreased the expression level of EN-related marker genes in the culture of human fetal PFC tissues.

To assess if the effects of FGF2 on human fetal cortex tissue culture are universal across different species, we also cultured mouse PFC cells with or without FGF2. Analysis of differentially expressed genes revealed that FGF2 administration significantly reduced the expression levels of EN marker genes, including *Glul*, *Camk2g*, and *Sla* in mouse primary culture cells (Figures 5G,H), consistent with the results in human fetal cortex tissue culture.

## 4. Discussion

Our scRNA-seq analysis revealed that NPC in the PFC tissues at different fetal stages have distinct potentials for differentiating into excitatory and inhibitory neurons. EN accounted for 68.1% of the cells in GW11, but this proportion significantly dropped to 13.6% in GW20, while IN cell types accounted for 5.4 and 49.3% of the total cells in GW11 and GW20, respectively. This shift was not due to bias induced by experiment procedures like dissociation, since the EN and IN marker gene expression levels were consistently significantly enhanced in the cultures of GW11 and GW20 PFC tissues, respectively. This trend is consistent with another study that reported a higher proportion of IN after 6 weeks of cell culture of PFC progenitors in GW18 compared to GW15 (Delgado et al., 2022). Interestingly, the cell ratios in primary GW11 and GW20 PFC tissues without cell culture (Zhong et al., 2018; Trevino et al., 2021) displayed the same higher EN and IN ratio in dissociated fetal PFC tissues at approximately GW11 and GW20 without cell culture, respectively. These results suggest that the progenitor cells in the PFC tissues at a given gestational stage have formed distinct cell identities and properties and differentiate into intrinsic cell types at inherent cell ratios. Our cell transition probability analysis revealed that this EN/IN ratio shift could be attributed to the intrinsic progenitor lineage specificity of the neural progenitor cells in PFC tissues at different gestation ages since the progenitor cells displayed a different expression profile between GW11 and GW20 samples. Therefore, the differential progenitor cells generate a higher proportion of IPC expressing *EOMES* that is differentiated into EN in the early-stage PFC cortex, but produce an increased proportion of inhibitory IPC progenitor cells in the late embryonic PFC, a trend that was further exacerbated under culture conditions. This finding is consistent with the progenitor lineage specificity mechanism that contributes to neural cell diversity (Franco et al., 2012). Moreover, the CGE/MGE marker genes *SCGN* and *NPY* absent in GW11 but present in most GW20 IN and IN precursor cells suggests the distinct cell sources of these cell types in the two samples, with the IN originating from the local neural progenitor cells in GW11, but mostly from CGE/MGE progenitors migrated into PFC region in GW20. Collectively these findings indicate that the proportions of neurons in the culture changed in the culture of PFC tissues at increasing gestation weeks due to their distinct progenitor lineage specificity and cell origins.

The availability of human brain tissue samples, especially those obtained by biopsy, is limited and poses a significant challenge to researchers (Lu et al., 2011). Although human-derived NPCs and various methods for inducing neuronal cells from human induced pluripotent stem cells (hiPSCs) are commercially available (Eichmuller and Knoblich, 2022), these methods do not fully recapitulate the true *in vivo* differentiation and development of primary NPCs. Moreover, the culture of hiPSCs usually requires the manipulation of sophisticated experimenters due to the complex experimental processes. In this study, we offered a simple-to-conduct and effective strategy for *in vitro* differentiation of human neural NPCs into EN or IN cells, which provides a valuable approach for experiments requiring large cell numbers. Compared to the previous protocols to culture brain tissues, our method simplified the experimental process (no need to conduct debris removal after the release of single cells from the digested fetal PFC tissues) and required fewer small molecule reagents (no need for FGF2 for EN culture), which not only reduces the reagent budge but also increases the success rate of obtaining high-quality primary cultured neuron cells (Brewer and Torricelli, 2007; Lu et al., 2011; Delgado et al., 2022). Importantly, our study provides researchers with a solution to obtain their desired cell type by selecting the PFC tissues of appropriate gestational ages for culture. For example, earlier gestational (GW11, GW13, and GW15) PFC tissues may be preferable for obtaining EN, whereas later gestational (GW20) PFC tissues may be more suitable for obtaining IN.

This report also investigated the function of FGF2, a reagent commonly added in the reported protocols of *in vitro* culture of neuronal cells and brain tissues (Woodbury and Ikezu, 2014). We found that the FGF2 administration tends to decrease the EN ratio in the culture of both human and mouse PFC tissues. Overall, our study offers researchers the possibility to optimize their neuron culture system.

Recent research has shown the capacity of human cortical progenitors to generate both EN and cortical interneurons under different *in vivo* and *in vitro* culture conditions (Delgado et al., 2022). Our study confirms these findings but is limited to elucidating the underlying mechanisms that regulate the abrupt EN-IN ratio shift in the *in vitro* cultures of human fetal PFC tissues at different gestation stages. Nevertheless, we report here an approach for *in vitro* culturing of neurons, which may be applied to generate EN and IN cells from patient models, providing a valuable tool to elucidate disease mechanisms at the cellular and molecular levels. This will facilitate the development of neuron transplantation therapies for the treatment of neurodegenerative diseases.

## Data availability statement

The bulk RNA-seq data of cultured mice P0 brain tissues have been deposited in the Gene Expression Omnibus (GEO) under the accession number GSE224114. The single-cell RNA-seq data and bulk RNA-seq data for the cultured human PFC tissues have been deposited in the Genome Sequence Archive (GSA) for Human under the accession number HRA003885 and HRA003880.

## Ethics statement

The studies involving human participants were reviewed and approved by the Reproductive Study Ethics Committee of Guangzhou Women and Children’s Medical Center (2021072816053867). Written informed consent to participate in this study was provided by the participants’ legal guardian/next of kin. And the use of C57BL/6 mice in this study was approved by the Animal Care and Use Committee of Sun Yat-sen University (No. SYSU-IACUC-2020-070).

## Author contributions

FF, XY, XW, and ZT conceived the ideas and supervised the project. WD and DX performed the bioinformatics analyses and revised the figures. RL, XW, and LH produced the bulk RNA-seq, scRNA-seq, and IHC results. RL applied for ethics approval, conducted experiments on human and mouse embryos, and wrote the manuscript. HZ and XPY collected the human tissue and provided technical support. XW and ZT proofread the manuscript. All authors contributed to the article and approved the submitted version.

## Funding

This research was supported by grants from the Science and Technology Program of Guangzhou (202007030001 and 202201020615).

## Conflict of interest

The authors declare that the research was conducted in the absence of any commercial or financial relationships that could be construed as a potential conflict of interest.

## Publisher’s note

All claims expressed in this article are solely those of the authors and do not necessarily represent those of their affiliated organizations, or those of the publisher, the editors and the reviewers. Any product that may be evaluated in this article, or claim that may be made by its manufacturer, is not guaranteed or endorsed by the publisher.

## Footnotes

^1^https://david.ncifcrf.gov/home.jsp

## References

[ref1] Anders S. Pyl P. T. Huber W. (2015). HTSeq--a Python framework to work with high-throughput sequencing data. Bioinformatics 31, 166–169. doi: 10.1093/bioinformatics/btu638, PMID: 25260700 PMC4287950

[ref2] Anderson S. A. Eisenstat D. D. Shi L. Rubenstein J. L. (1997). Interneuron migration from basal forebrain to neocortex: dependence on dlx genes. Science 278, 474–476. doi: 10.1126/science.278.5337.474, PMID: 9334308

[ref3] Anderson S. A. Marin O. Horn C. Jennings K. Rubenstein J. L. R. (2001). Distinct cortical migrations from the medial and lateral ganglionic eminences. Development 128, 353–363. doi: 10.1242/dev.128.3.353, PMID: 11152634

[ref4] Brewer G. J. Torricelli J. R. (2007). Isolation and culture of adult neurons and neurospheres. Nat. Protoc. 2, 1490–1498. doi: 10.1038/nprot.2007.207 17545985

[ref5] De Bruyckere E. Simon R. Nestel S. Heimrich B. Kätzel D. Egorov A. V.. (2018). Stability and function of hippocampal mossy Fiber synapses depend on Bcl11b/Ctip2. Front. Mol. Neurosci. 11:103. doi: 10.3389/fnmol.2018.00103, PMID: 29674952 PMC5895709

[ref6] de Guglielmo G. Iemolo A. Nur A. Turner A. Montilla-Perez P. Martinez A.. (2022). Reelin deficiency exacerbates cocaine-induced hyperlocomotion by enhan cing neuronal activity in the dorsomedial striatum. Genes Brain Behav. 21:e12828. doi: 10.1111/gbb.12828, PMID: 35906757 PMC9744517

[ref7] Delgado R. N. Allen D. E. Keefe M. G. Leon W. R. M. Ziffra R. S. Crouch E. E.. (2022). Individual human cortical progenitors can produce excitatory and inhibitory neurons. Nature 601, 397–403. doi: 10.1038/s41586-021-04230-7, PMID: 34912114 PMC8994470

[ref8] Eichmuller O. L. Knoblich J. A. (2022). Human cerebral organoids - a new tool for clinical neurology research. Nat. Rev. Neurol. 18, 661–680. doi: 10.1038/s41582-022-00723-9, PMID: 36253568 PMC9576133

[ref9] Farrell J. A. Wang Y. Riesenfeld S. J. Shekhar K. Regev A. Schier A. F. (2018). Single-cell reconstruction of developmental trajectories during zebrafish embryogenesis. Science 360:eaar3131. doi: 10.1126/science.aar3131 29700225 PMC6247916

[ref10] Finak G. McDavid A. Yajima M. Deng J. Y. Gersuk V. Shalek A. K.. (2015). MAST: a flexible statistical framework for assessing transcriptional changes and characterizing heterogeneity in single-cell RNA sequencing data. Genome Biol. 16:278. doi: 10.1186/s13059-015-0844-5 26653891 PMC4676162

[ref11] Franco S. J. Gil-Sanz C. Martinez-Garay I. Espinosa A. Harkins-Perry S. R. Ramos C.. (2012). Fate-restricted neural progenitors in the mammalian cerebral cortex. Science 337, 746–749. doi: 10.1126/science.1223616, PMID: 22879516 PMC4287277

[ref12] Gorski J. A. Talley T. Qiu M. Puelles L. Rubenstein J. L. R. Jones K. R. (2002). Cortical excitatory neurons and glia, but not GABAergic neurons, are produced in the Emx1-expressing lineage. J. Neurosci. 22, 6309–6314. doi: 10.1523/JNEUROSCI.22-15-06309.2002 12151506 PMC6758181

[ref13] Gupta S. M-Redmond T. Meng F. Tidball A. Akil H. Watson S.. (2018). Fibroblast growth factor 2 regulates activity and gene expression of human post-mitotic excitatory neurons. J. Neurochem. 145, 188–203. doi: 10.1111/jnc.14255, PMID: 29168882 PMC5924590

[ref14] Hao Y. H. Hao S. Andersen-Nissen E. Mauck W. M. Zheng S. W. Butler A.. (2021). Integrated analysis of multimodal single-cell data. Cells 184:3573-+. doi: 10.1016/j.cell.2021.04.048, PMID: 34062119 PMC8238499

[ref15] Hayashida M. Hashioka S. Hayashida K. Miura S. Tsuchie K. Araki T.. (2020). Low serum levels of fibroblast growth factor 2 in Gunn rats: a hyper-bilirubinemia animal model of schizophrenic symptoms. CNS Neurol. Disord. Drug Targets 19, 503–508. doi: 10.2174/1871527319999200729153907, PMID: 32729434

[ref16] Huang da W. Sherman B. T. Lempicki R. A. (2009). Systematic and integrative analysis of large gene lists using DAVID bioinformatics resources. Nat. Protoc. 4, 44–57. doi: 10.1038/nprot.2008.211, PMID: 19131956

[ref17] Ji Z.-S. Liu Q.-L. Zhang J.-F. Yang Y.-H. Li J. Zhang G.-W.. (2020). SUMOylation of spastin promotes the internalization of GluA1 and regul ates dendritic spine morphology by targeting microtubule dynamics. Neurobiol. Dis. 146:105133. doi: 10.1016/j.nbd.2020.105133, PMID: 33049318

[ref18] Kato A. C. Touzeau G. Bertrand D. Bader C. R. (1985). Human spinal cord neurons in dissociated monolayer cultures: morphological, biochemical, and electrophysiological properties. J. Neurosci. 5, 2750–2761. doi: 10.1523/JNEUROSCI.05-10-02750.1985, PMID: 2413186 PMC6565137

[ref19] Kelley K. W. Pasca S. P. (2022). Human brain organogenesis: toward a cellular understanding of development and disease. Cells 185, 42–61. doi: 10.1016/j.cell.2021.10.003, PMID: 34774127 PMC13523617

[ref20] Kessaris N. Magno L. Rubin A. N. Oliveira M. G. (2014). Genetic programs controlling cortical interneuron fate. Curr. Opin. Neurobiol. 26, 79–87. doi: 10.1016/j.conb.2013.12.012, PMID: 24440413 PMC4082532

[ref21] Kim E. J. Hori K. Wyckoff A. Dickel L. K. Koundakjian E. J. Goodrich L. V.. (2011). Spatiotemporal fate map of neurogenin1 (Neurog1) lineages in the mouse central nervous system. J. Comp. Neurol. 519, 1355–1370. doi: 10.1002/cne.22574, PMID: 21452201 PMC4010191

[ref22] Kim D. Paggi J. M. Park C. Bennett C. Salzberg S. L. (2019). Graph-based genome alignment and genotyping with HISAT2 and HISAT-genotype. Nat. Biotechnol. 37, 907–915. doi: 10.1038/s41587-019-0201-4, PMID: 31375807 PMC7605509

[ref23] Korsunsky I. Millard N. Fan J. Slowikowski K. Zhang F. Wei K.. (2019). Fast, sensitive and accurate integration of single-cell data with harmony. Nat. Methods 16, 1289–1296. doi: 10.1038/s41592-019-0619-0, PMID: 31740819 PMC6884693

[ref24] Li H. Handsaker B. Wysoker A. Fennell T. Ruan J. Homer N.. (2009). The sequence alignment/map format and SAMtools. Bioinformatics 25, 2078–2079. doi: 10.1093/bioinformatics/btp352, PMID: 19505943 PMC2723002

[ref25] Love M. I. Huber W. Anders S. (2014). Moderated estimation of fold change and dispersion for RNA-seq data with DESeq2. Genome Biol. 15:550. doi: 10.1186/s13059-014-0550-8, PMID: 25516281 PMC4302049

[ref26] Lu J. Delli-Bov L. C. Hecht J. Folkerth R. Sheen V. L. (2011). Generation of neural stem cells from discarded human fetal cortical tissue. J. Vis. Exp. 10, 2681–3791. doi: 10.3791/2681 PMC3197109 21654623

[ref27] Martinez-Morales P. L. Quiroga A. C. Barbas J. A. Morales A. V. (2010). SOX5 controls cell cycle progression in neural progenitors by interfer ing with the WNT-beta-catenin pathway. EMBO Rep. 11, 466–472. doi: 10.1038/embor.2010.61, PMID: 20448664 PMC2892326

[ref28] McGinnis C. S. Murrow L. M. Gartner Z. J. (2019). DoubletFinder: doublet detection in single-cell RNA sequencing data using artificial nearest neighbors. Cell Syst. 8, 329–337.e4. doi: 10.1016/j.cels.2019.03.003, PMID: 30954475 PMC6853612

[ref29] Nogueira A. B. Hoshino H. S. R. Ortega N. C. dos Santos B. G. S. Teixeira M. J. (2022). Adult human neurogenesis: early studies clarify recent controversies and go further. Metab. Brain Dis. 37, 153–172. doi: 10.1007/s11011-021-00864-8, PMID: 34739659

[ref30] Nowakowski T. J. Bhaduri A. Pollen A. A. Alvarado B. Mostajo-Radji M. A. Di Lullo E.. (2017). Spatiotemporal gene expression trajectories reveal developmental hierarchies of the human cortex. Science 358, 1318–1323. doi: 10.1126/science.aap8809, PMID: 29217575 PMC5991609

[ref31] Qian X. Davis A. A. Goderie S. K. Temple S. (1997). FGF2 concentration regulates the generation of neurons and glia from multipotent cortical stem cells. Neuron 18, 81–93. doi: 10.1016/s0896-6273(01)80048-9, PMID: 9010207

[ref32] Recabal A. Fernandez P. Lopez S. Barahona M. J. Ordenes P. Palma A.. (2021). The FGF2-induced tanycyte proliferation involves a connexin 43 hemichannel/purinergic-dependent pathway. J. Neurochem. 156, 182–199. doi: 10.1111/jnc.15188, PMID: 32936929 PMC7894481

[ref33] Sah D. W. Y. (1995). Human fetal central neurons in culture: voltage- and ligand-gated current. J. Neurophysiol. 74, 1889–1899. doi: 10.1152/jn.1995.74.5.1889, PMID: 8592182

[ref34] Santos-Gómez A. Miguez-Cabello F. García-Recio A. Locubiche-Serra S. García-Díaz R. Soto-Insuga V.. (2020). Disease-associated GRIN protein truncating variants trigger NMDA recep tor loss-of-function. Hum. Mol. Genet. 29, 3859–3871. doi: 10.1093/hmg/ddaa220 33043365

[ref35] Sherman B. T. Hao M. Qiu J. Jiao X. Baseler M. W. Lane H. C.. (2022). DAVID: a web server for functional enrichment analysis and functional annotation of gene lists (2021 update). Nucleic Acids Res. 50, W216–W221. doi: 10.1093/nar/gkac194, PMID: 35325185 PMC9252805

[ref36] Sudarov A. Turnbull R. K. Kim E. J. Lebel-Potter M. Guillemot F. Joyner A. L. (2011). Ascl1 genetics reveals insights into cerebellum local circuit assembly. Dev. Biol. 356:238. doi: 10.1016/j.ydbio.2011.05.414 PMC3153985 21795554

[ref37] Telley L. Jabaudon D. (2018). A mixed model of neuronal diversity. Nature 555, 452–454. doi: 10.1038/d41586-018-02539-4 32034370

[ref38] Trevino A. E. Müller F. Andersen J. Sundaram L. Kathiria A. Shcherbina A.. (2021). Chromatin and gene-regulatory dynamics of the developing human cerebra l cortex at single-cell resolution. Cells 184, 5053–5069.e23. doi: 10.1016/j.cell.2021.07.039, PMID: 34390642

[ref39] van der Maaten L. Hinton G. (2008). Visualizing data using t-SNE. J. Mach. Learn. Res. 9, 2579–2605.

[ref40] Wang H. Ge G. Uchida Y. Luu B. Ahn S. (2011). Gli3 is required for maintenance and fate specification of cortical pr ogenitors. J. Neurosci. 31, 6440–6448. doi: 10.1523/JNEUROSCI.4892-10.2011, PMID: 21525285 PMC3096934

[ref41] Woodbury M. E. Ikezu T. (2014). Fibroblast growth Factor-2 signaling in neurogenesis and neurodegeneration. J. Neuroimmune Pharmacol. 9, 92–101. doi: 10.1007/s11481-013-9501-5, PMID: 24057103 PMC4109802

[ref42] Young M. D. Behjati S. (2020). SoupX removes ambient RNA contamination from droplet-based single-cell RNA sequencing data. Gigascience 9:giaa151. doi: 10.1093/gigascience/giaa151 33367645 PMC7763177

[ref43] Zhong S. J. Zhang S. Fan X. Y. Wu Q. Yan L. Y. Dong J.. (2018). A single-cell RNA-seq survey of the developmental landscape of the human prefrontal cortex. Nature 555:524-+. doi: 10.1038/nature25980, PMID: 29539641

[ref44] Ziffra R. S. Kim C. N. Ross J. M. Wilfert A. Turner T. N. Haeussler M.. (2021). Single-cell epigenomics reveals mechanisms of human cortical developme nt. Nature 598, 205–213. doi: 10.1038/s41586-021-03209-8, PMID: 34616060 PMC8494642

