# Supplementary material for: Single-cell RNA-sequencing identifies various proportions of excitatory and inhibitory neurons in cultured human fetal brain cortical tissues
Source: Front Neurosci. 2023 Jun 28;17:1177747. doi: 10.3389/fnins.2023.1177747 (PMC10338112; doi:10.3389/fnins.2023.1177747)
Supplement: Supplementary file 1 [file Data_Sheet_1.docx]

Single-cell RNA-sequencing identifies various proportions of excitatory and inhibitory neurons in cultured human fetal brain cortical tissues

Rong Liu^1^, Wei Dong^1^, Dan Xiong^1^, Zhonghui Tang ^1^, Lanqi Hu^1^, Haoran Zhang^1^, Xiaoping Yuan^2^, Fang Fu^2*^, Xin Yang^2*^, Xia Wu ^1*^.

^1^ Zhongshan School of Medicine, Sun Yat-sen University, Guangzhou, Guangdong, China

^2^ Department of Prenatal Diagnostic Center, Guangzhou Women and Children’s Medical Center, Guangzhou Medical University, Guangzhou, Guangdong, China

* **Correspondence:**

Fang Fu: fuyingyi2008@163.com

Xin Yang: yangsunrise@163.com

Xia Wu: wuxia23@mail.sysu.edu.cn

**Supplementary Figures**


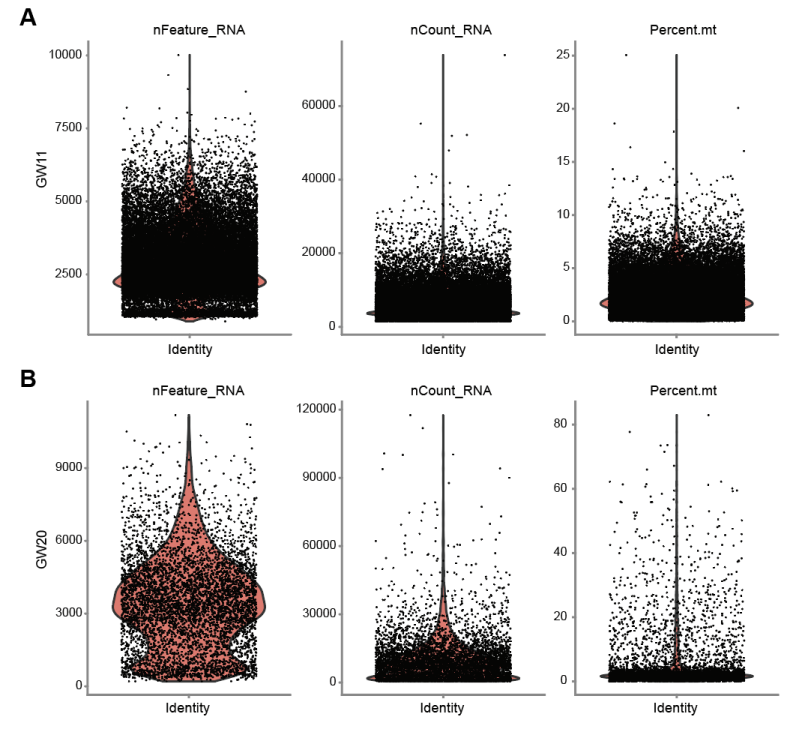


**Supplementary Figure 1.**

Violin plots showing the key quality control parameters for the GW11 (**A**) and GW20 (**B**) datasets. The nFeature_RNA and nCount_RNA represent the numbers of genes and read counts detected per cell, respectively, while the Percent.mt represents the percentage of read counts from mitochondrial genes.
